# Precise Localization in Craniotomy With a Retrosigmoid Keyhole Approach: Microsurgical Anatomy and Clinical Study

**DOI:** 10.3389/fsurg.2022.809098

**Published:** 2022-04-14

**Authors:** Zhi-Heng Jian, Min-Feng Sheng, Jia-Yan Li, Yu Li, Zhi-Jian Weng, Gang Chen

**Affiliations:** ^1^Department of Neurosurgery, Zhuhai People's Hospital, Jinan University, Zhuhai, China; ^2^Department of Neurosurgery, Second Affiliated Hospital, Soochow University, Suzhou, China

**Keywords:** suboccipital retrosigmoid approach, craniotomy, mastoid groove, transverse sinus, sigmoid sinus

## Abstract

**Objective:**

We aimed to explore a method of precise localization within craniotomy based on skull anatomical landmarks via the suboccipital retrosigmoid approach.

**Method:**

Craniometric measurements were taken from 15 adult dry skulls and eight cadaver head specimens. In the anatomical study, the keypoint corresponded to the transverse-sigmoid sinus junction's corresponding point on the external surface of the temporal mastoid process, eight cadaveric heads underwent a simulated craniotomy using the suboccipital retrosigmoid approach. The center of the burr hole is precisely oriented 12 mm vertically above the top point of the mastoid groove based on the line between the infraorbital margin and the upper edge of the external auditory canal. Clinical application was verified in clinical surgery by evaluating the accuracy, safety, rapidity, and minimal invasiveness of the procedure in 29 patients.

**Result:**

No venous sinus injuries were observed. Within clinical application, 29 patients underwent craniotomy using the suboccipital retrosigmoid approach. The operative area was clearly exposed in all patients and the microsurgical anatomy of the intracranial region after the dura mater incision was satisfactory. No venous sinus ruptures were observed. The average craniectomy time was 27.02 ± 0.86 min. The diameter of the bone window was 1.7–2.9 cm.

**Conclusion:**

We conclude that the method can ensure safe, accurate, and rapid craniotomy with good vision while avoiding injury to the venous sinus.

## Introduction

The suboccipital retrosigmoid approach is often used to treat lesions in the cerebellopontine angle regions. Full exposure of the inferior margin of the transverse sinus, the posterior margin of the sigmoid sinus, and the transverse-sigmoid sinus junction enlarge the operation fields and decrease the likelihood of injuries caused by distraction of the brain tissue. Therefore, it is necessary to precisely select the proper, optimal keypoint for craniotomy. Landmarks, such as the asterion, the superior nuchal line, the line between the external occipital protuberance and the root of the zygomatic arch, and the posterior margin of the root of the mastoid, are used to locate important intracranial structures (1). The asterion is considered to be an external superficial landmark corresponding to the transverse-sigmoid sinus junction (2). Nevertheless, a few reports have described anatomic variations at this point (3, 4). Thus far, many reports have proposed a series of methods using superficial landmarks and/or radiological technology (5–7). However, the application of their proposed methods has been restricted by complicated processes and inaccurate locations. Hence, we developed an improved method of craniotomy via analyzing the relationships between superficial landmarks on adult skulls (8). In this study we verified the feasibility of this method in a clinical setting.

## Methods

### Specimens and Instruments

Twenty-three cadaveric specimens (15 adult skulls and eight cadaveric head specimens) collected from the Human Anatomy Center of Soochow University were used in this study. No obvious deformities were observed in the posterior cranial fossa, petrous, or mastoid parts of the temporal and occipital bones. A Zeiss microscope (OPMI PROergo, Germany), a SNAKE Microspeed Uni Dynamical System (GA B20 DBP, Germany), homemade head racks, microinstruments, and a scaleplate (accuracy: 1 mm) were used in the current study.

A total of 29 patients were enrolled in this study. The enrolled patients underwent accurate craniotomy with a retrosigmoid approach. All patients were operated by one senior neurosurgeon. All patients were enrolled within Zhuhai People's Hospital from October 2019 to December 2020 (six males, 23 females; ages 32–66 years, mean age: 50.79 ± 19.80 years). Seven patients were diagnosed with acoustic neuroma, four were diagnosed with trigeminal neuralgia, two were diagnosed with meningioma, 14 were diagnosed with hemifacial spasm, one was diagnosed with schwannoma of the jugular foramen area, and one patient was diagnosed with cholesteatoma.

The studies involving human participants were reviewed and approved by the Ethics Committee of Zhuhai People's Hospital. The patients/participants provided written informed consent prior to participating in this study.

### Accurate Craniotomy of the Retrosigmoid Approach in Cadaveric Specimens

Based on the relationships among landmarks on the skull, we developed a safe, accurate, and rapid method of craniotomy, in which we modified the retrosigmoid approach in cadaveric specimens and evaluated the results in an anatomical study.

**Figure 1:**
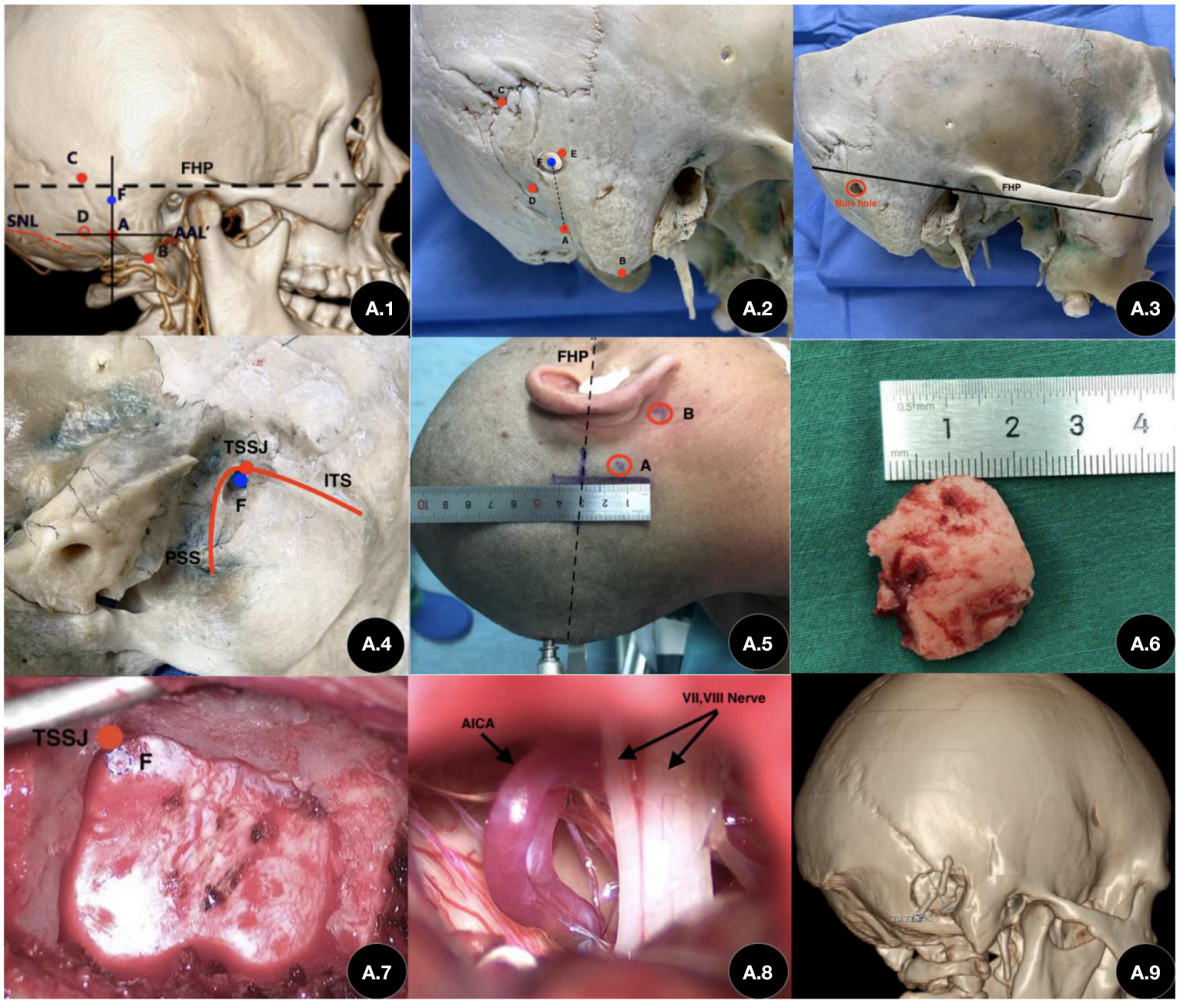
Application of our method in craniotomy with a retrosigmoid keyhole approach. **(A.1)** Preoperative three-dimensional reconstructed images and related anatomical landmarks. **(A.2)** The center of burr hole, located approximately 12 mm superior to the top point of the mastoid groove on the vertical line (A–F). **(A.3)** The Frankfurt horizontal plane (FHP) and the burr hole. **(A.4)** Inner view of the transverse sinus–posterior margin of the sigmoid sinus junction (TSSJ) and the center point of the burr hole. **(A.5)** Intraoperative incision (~4 cm). **(A.6)** Size of the bone flap. **(A.7)** Exposure of the bone window during the operation. **(A.8)** Exposure during MVD surgery. **(A.9)** Three-dimensional reconstruction images after the operation. (A) Top point of the mastoid groove. (B) Mastoid apex. (C) Asterion. (D) Foramen venosum of the mastoid emissary vein. (E) Keypoint (the correspondence point to the transverse-sigmoid sinus junction on the external surface of the skull). (F) Center point of the burr hole. (FHP) Linen parallel line to the line between the infraorbital margin and the upper edge of the external auditory canal. (TSSJ) Transverse-Sigmoid Sinus Junction. (PSS) Posterior edge of Sigmoid Sinus. (ITS) Inferior edge of Transeverse Sinus.

The bony anatomical structures were marked on the external surface of the skulls as follows (Figures 1A.2–A.4): the top point of the digastric groove (A), the mastoidale (B), the asterion (C), the center of the burr-hole (F), and the Frankfurt horizontal plane (FHP). Next, on the inner surface of the dry skull, we identified the inferior margin of the transverse sinus (ITS) and the posterior margin of the sigmoid sinus (PSS), subsequently marking the inferior margin of the transverse sinus–posterior margin of the sigmoid sinus junction (TSSJ). We then drilled the skull vertically from the inside to the outside on the basis of the TSSJ. The TSSJ was re-confirmed on the inner surface of the skulls. The corresponding point on the external surface of the skulls served as the keypoint (E). Following this, the lengths were measured between the keypoint and the digastric groove (AE); these were similar to the lengths between the keypoint and mastoidale (BE) and between the keypoint and the asterion (CE). (Finally, a method for precisely locating the keypoint in the retrosigmoid keyhole approach was developed according to the findings for the dry skulls. Specifically, eight cadaveric heads underwent a simulated craniotomy using the suboccipital retrosigmoid approach. No venous sinus injuries were observed. The centers of the burr holes were found to be posterior (inferior to the transverse sigmoid sinus).

### Accurate Craniotomy: Retrosigmoid Approach Based on the Anatomic Study

All patients underwent thin-slice computed tomography (CT) and three-dimensional reconstruction of the skull prior to surgery. We evaluated the landmarks in the reconstructed images and created a plan for craniotomy (Figure 1A.1). All surgeries were performed by an experienced neurosurgeon. After the patient was anesthetized, we located the root and apex of the mastoid. We then marked the straight incision posterior to the ears, as well as the line between the infraorbital margin and the upper edge of the external auditory canal (Figure 1A.5). Next, we exposed the posterior margin of the mastoid and the top point of the mastoid groove. We drew a vertical line at the top point of the mastoid groove based on the line between the infraorbital margin and the upper edge of the external auditory canal which is consistent to the Frankurt Horizontal Plane. We then drilled the burr holes with a semi-diameter of 3 mm and formed the bone flap using a milling cutter (Figure 1A.7). We reserved some bones near the mastoidal emissary veins and exposed the posterior margin of the sigmoid sinus and the inferior margin of the transverse sinus. We sealed the posterior margin of the mastoid using bone wax. The bone was restored after the craniotomies were completed. After the operation, a thin-slice CT scan and three-dimensional reconstruction were performed again in order to verify the accuracy of the method (Figure 1A.9).

### Clinical Criteria

With respect to accuracy, the local anatomic structure was precisely located and fully exposed. With respect to safety, we evaluated whether there was any injury to locally important anatomic structures during craniotomy. For evaluating rapidity, the time duration for the craniotomy was calculated using the incision to ensure exposure of the bone window. Finally, with regard to micro-invasion, we evaluated the size of the bone flap and the bone window as well as whether or not the bone flap was restored.

### Statistical Analysis

The results were expressed as $\bar{x}\pm s$. Student's *t*-test was used to perform statistical comparisons via the Statistical Package for Social Sciences (SPSS) for Windows (version 21.0, Chicago, IL, US) with statistical significance set at a *p*-value of <0.05.

## Results

### Skull Studies

The distances from keypoint to the top point of mastoid groove were 16.80 ± 0.61 mm (left) and 14.83 ± 5.13 mm (right). The distances from the keypoint to the asterion were 19.03 ± 2.56 mm (left) and 22.60 ± 4.15 mm (right). The distances from the keypoint to the mastoidale were 33.99 ± 4.16 mm (left) and 32.78 ± 6.40 mm (right). The value of the left distance from the keypoint to the asterion was smaller than that on the right side (Table 1). Therefore, we developed the location method described previously. The center of the burr hole was precisely oriented at 12 mm above the top point of the mastoid groove vertically based on the Frankurt Horizontal Plane. As previously described, eight cadaveric heads underwent simulated craniotomy via the suboccipital retrosigmoid approach. No venous sinus injuries were observed. The centers of the burr holes were all posterior (inferior to the transverse sigmoid sinus junction).

**Table 1:** The length between anatomical landmarks in dry skulls (mm).

	**AE**	**BE**	**CE**
**Left**	16.80 ± 0.61	33.99 ± 4.16	19.03 ± 2.56
**Right**	14.83 ± 5.13	32.78 ± 6.40	22.60 ± 4.15
***t***	−1.663	−0.917	2.468
***P***	0.107	0.367	0.020

*AE, lengths measured between the keypoint and digastric groove; BE, lengths between the keypoint and mastoidale; CE, lengths between the keypoint and asterion*.

### Clinical Applications

With respect to accuracy, 29 patients underwent the previously described operations in order to verify the accuracy, safety, and rapidity of our proposed methodology (Table 2). The transverse and sigmoid sinuses of 26 of the patients were well exposed which the distance of center of burr holes and TSSJ were exactly 3 mm. Except 3 patients were reserved some bone.The sinuses of the remaining three patients were also well exposed following adequate bone grinding. The local microanatomic structure of the operation fields was well exposed after the dura was cut (Figure 1A.8).

**Table 2:** Clinical data for the 29 enrolled patients.

**Patient**	**Gender**	**Age**	**Diagnosis**	**Bone window (mm)**	**Bone flap (mm)**	**Craniectomy time (min)**
^***^	F	36	Cholesteatoma	29^*^25	15^*^18	23.54
^***^	F	38	HFS	26^*^25	21^*^20	33.43
^***^	F	41	HFS	23^*^21	18^*^17	27.59
^***^	M	55	HFS	25^*^21	23^*^17	30.12
^***^	M	43	HFS	27^*^20	22^*^19	24.33
^***^	M	44	HFS	28^*^25	21^*^22	23.22
^***^	F	52	HFS	19^*^20	15^*^19	31.22
^***^	F	32	HFS	17^*^22	14^*^18	23.23
^***^	M	50	HFS	27^*^22	24^*^17	22.12
^***^	F	34	HFS	25^*^23	23^*^21	31.22
^***^	F	57	HFS	16^*^25	13^*^23	33.23
^***^	F	58	HFS	27^*^20	12^*^17	22.35
^***^	F	64	HFS	25^*^21	14^*^18	33.42
^***^	F	59	HFS	23^*^19	15^*^16	25.22
^***^	F	66	HFS	25^*^20	14^*^15	27.43
^***^	F	59	Meningeoma	27^*^23	18^*^17	23.33
^***^	F	47	Meningeoma	22^*^21	19^*^16	35.21
^***^	M	58	TN	20^*^25	18^*^23	31.53
^***^	F	63	TN	21^*^22	13^*^19	26.38
^***^	F	61	TN	20^*^16	15^*^14	25.23
^***^	M	50	TN	27^*^20	17^*^18	21.32
^***^	F	46	Schwannoma	27^*^24	15^*^15	25.26
^***^	F	37	Acoustic Neuroma	25^*^22	15^*^17	25.21
^***^	F	38	Acoustic Neuroma	26^*^28	18^*^24	24.33
^***^	F	53	Acoustic Neuroma	24^*^25	17^*^15	27.57
^***^	M	59	Acoustic Neuroma	22^*^20	12^*^15	28.48
^***^	F	54	Acoustic Neuroma	27^*^25	23^*^20	29.52
^***^	F	55	Acoustic Neuroma	23^*^20	14^*^16	26.27
^***^	F	64	Acoustic Neuroma	26^*^25	15^*^20	22.32

*HFS, Hemifacial Spasm; TN, Trigeminal Neuralgia*.

With regard to safety, our method was applied to 29 patients and no injury to the venous sinuses caused by drilling was observed. The sigmoid sinus of one patient was injured because of poor handling of the emissary veins during the formation of the bone flap. The injury was controlled and minimized immediately and had no relationship with the location method.

For evaluating rapidity, the mean craniotomy time was 27.02 ± 0.86 min. Finally, with respect to micro-invasion, the diameter of the bone flap was 1.2–2.4 cm (Figure 1A.6) and the mean diameter of the bone windows ranged from 1.7 to 2.9 cm. The bone flap was restored during the surgery.

## Discussion

The retrosigmoid approach is widely used in neurosurgery. This approach exposes important structures in the cerebellopontine angle regions using the gap between the cerebellum and the petrosal bone. Since the space is restricted by the transverse and sigmoid sinuses, it is necessary to fully expose the inferior margin of the transverse sinus, the posterior margin of the sigmoid sinus, and the transverse-sigmoid sinus junction. Therefore, precise identification of the correspondence point with respect to the transverse-sigmoid sinus junction on the external surface of the skull can ensure the best exposure and thus lead to precise, safe, and effective craniotomy (9).

The development of radiological technology provides many new and precise methods for locating the keypoint (10, 11). Specifically, radiological technology can simultaneously detect anatomic variations and inform the formulation of an effective approach for minimizing complications and injuries. Nevertheless, these methods are restricted by the availability of equipment and specialists as well as by logistics surrounding expenses and preoperative preparations. Any error will lead to serious consequences during operation. Therefore, it is crucial to find a convenient and accurate method for locating the keypoint and guiding accurate craniotomy. In the current study, we evaluated optimal surgical methodology given these considerations by implementing and investigating the retrosigmoid approach.

Superficial landmarks, such as the asterion, the superior nuchal line, the line between the external occipital protuberance and the root of the zygomatic arch, and the posterior margin of the root of the mastoid, are used to locate important intracranial structures. As previously mentioned, the asterion is generally considered an external superficial landmark corresponding to the transverse-sigmoid sinus junction (12–15). A few reports describe anatomic variations in asterions (16–18). For example, approximately 85% of the asterions are located precisely at the lateral side of the transverse sinus, ~10% of the asterions are located above the transverse sinus, and ~5% are located below the transverse sinus.The asterion is often posteriorly superior to the keypoint. Therefore, there may be a risk of injuring the venous sinus when using asterion as the keypoint within craniotomy.

Anatomic variations were observed in the mastoid foramen. The mean diameter of the mastoid emissary vein foramen was 2.15 ± 0.81 mm occurring at a rate of 61%. The number of mastoid emissary veins foramen ranged from 1 to 4.However, it is consistently located anterior to the occipitomastoid suture (19). The significantly high rate of variability of the mastoid foramen leads to difficulty in precisely locating the keypoint. However, the keypoint is always anterosuperior to the mastoid foramen. Further modifications were made after locating the keypoint according to the digastric groove and mastoidale to precisely locate the keypoint in its anterosuperior relation to the mastoid foramen. The lateral part of the SNL is located below the level of the transverse sinus and the length between the SNL and transverse sinus ranges from 1.5 mm to 14 mm (20). The high variation rate of the relationship of SNL and transverse sinus is also inaccurate for locating the keypoint. However, the keypoint is always mildly above the lateral portion of the SNL (21). Subsequently, further modifications were made according to the relationship between the venous sinus and mastoid foramen/SNL. These modifications also provide the locating information of the keypoint: anterosuperior to the mastoid foramen, above the lateral portion of the SNL.

Based on a detailed study of skull specimens, the current research proposes a new theory that uses the top point of the mastoid groove as a reference point for locating the keypoint. Based on the line between the infraorbital margin and the upper edge of the external auditory canal, we drew a vertical line at the top point of the mastoid groove, which was approximately 12 mm superior to the top point of the mastoid groove on the vertical line, and formed a burr hole with a semi-diameter of 3 mm at this point. We verified our method in 29 patients. Our results showed that the accuracy, safety, rapidity, and micro-invasion of this neurosurgical methodology will guarantee wide application in clinical studies. After the operation, we reconstructed the skull and bone windows using a three-dimensional CT technique. We observed that the keypoint was at the anterior (inferior to the asterion), and that its location was consistent with previous anatomical studies. Therefore, we demonstrated that the proposed methodology was accurate during surgery.

In addition to the substantial strengths of this investigation, we also acknowledge several limitations. First, the small number of enrolled participants and anatomical specimens and the unknown age and sex of the skulls may interfere with the generalizability, preciseness, and general interpretation of our results. Second, variation is inevitable when applying this method to high-powered, robust, gold-standard clinical studies. Therefore, further studies are needed to validate this methodology prior to issuing recommendations for surgical guidelines.Considering variations for individual patients and the complex considerations involved in issuing surgical recommendations, we will verify the accuracy of this methodology using guiding systems in future, high-powered, gold-standard clinical studies.

## Conclusion

The findings of the current study demonstrate that in order to precisely locate the keypoint within craniotomy via the suboccipital retrosigmoid approach, the center of the burr hole can be precisely oriented (12 mm vertically above the top point of the mastoid groove) based on the line between the infraorbital margin and the upper edge of the external auditory canal. Although our findings need to be validated in future studies, our results show that the proposed methodology could ensure safe, accurate, and rapid craniotomy with good vision while avoiding injury to the vein sinuses.

## Data Availability Statement

The original contributions presented in the study are included in the article/Supplementary Material, further inquiries can be directed to the corresponding author.

## Ethics Statement

The studies involving human participants were reviewed and approved by the Ethics Committee of Zhuhai People's Hospital. The patients/participants provided their written informed consent to participate in this study.

## Author Contributions

GC contributed to the study concept and design. M-FS, J-YL, and YL contributed to the acquisition of data. Z-JW contributed to the analysis and interpretation of data. Z-HJ contributed to the drafting of the manuscript. All authors read and approved the final manuscript.

## Funding

This work was supported by Soochow Key Health Talents Project of Jiangsu province, 2014 (to GC) and Zhuhai People's Hospital Scientific Research Initiation Project No. 2021KYQD-02 (to GC).

## Conflict of Interest

The authors declare that the research was conducted in the absence of any commercial or financial relationships that could be construed as a potential conflict of interest.

## Publisher's Note

All claims expressed in this article are solely those of the authors and do not necessarily represent those of their affiliated organizations, or those of the publisher, the editors and the reviewers. Any product that may be evaluated in this article, or claim that may be made by its manufacturer, is not guaranteed or endorsed by the publisher.
